# The roles of heme oxygenase-1 in renal disease

**DOI:** 10.3389/fneph.2023.1156346

**Published:** 2023-05-05

**Authors:** Hongfu Zhai, Lihua Ni, Xiaoyan Wu

**Affiliations:** ^1^Department of Nephrology, Zhongnan Hospital of Wuhan University, Wuhan, China; ^2^Department of General Practice, Zhongnan Hospital of Wuhan University, Wuhan, China

**Keywords:** heme oxygenase, renal disease, mechanism, value, therapeutic

## Abstract

Heme oxygenase (HO), a heat shock protein containing hemoglobin, is an important enzyme in heme catabolism. It is involved in cell homeostasis and has anti-inflammatory, antioxidant, anti-apoptosis, immunomodulation, and other functions. It is expressed at a modest level in most normal tissues. When the body suffers from ischemia hypoxia, injury, toxins, and other nociceptive stimuli, the expression increases, which can transform the oxidative microenvironment into an antioxidant environment to promote tissue recovery from damage. In recent years, research has continued to verify its value in a variety of human bodily systems. It is also regarded as a key target for the treatment of numerous disorders. With the advancement of studies, its significance in renal disease has gained increasing attention. It is thought to have a significant protective function in preventing acute kidney injury and delaying the progression of chronic renal diseases. Its protective mechanisms include anti-inflammatory, antioxidant, cell cycle regulation, apoptosis inhibition, hemodynamic regulation, and other aspects, which have been demonstrated in diverse animal models. Furthermore, as a protective factor, its potential therapeutic efficacy in renal disease has recently become a hot area of research. Although a large number of preclinical trials have confirmed its therapeutic potential in reducing kidney injury, due to the problems and side effects of HO-1 induction therapy, its efficacy and safety in clinical application need to be further explored. In this review, we summarize the current state of research on the mechanism, location, and treatment of HO and its relationship with various renal diseases.

## 1 Overview of HO

HO is an important enzyme in heme degradation and metabolism, and can decompose heme into three metabolites: biliverdin IX, CO, and Fe^2+^. It includes three distinct active subtypes, namely oxygen stress inducible (HO-1), constitutive heme oxygenase (HO-2) and HO-3, which have not yet been fully described, and was first found by Tenhunen and his colleagues in 1968 (1). Among these subtypes, HO-1 has the most biological activity and is the most frequently studied. It plays a significant role in cell stress and the maintenance of cell homeostasis, which is linked to numerous clinical disease states (2). Heavy metals, endotoxin, UV, cytokines, growth hormones, and other chemical and physical stimuli can stimulate HO-1 expression, which in turn promotes the generation of antioxidants and anti-apoptosis molecules to protect cells. It also promotes injury repair through immunity, regulation of cell autophagy, inhibition of apoptosis, and the regulation of cell cycle and other pathways. Nath and colleagues first found its kidney-protective effect in a rat model of glycerol-induced hemolytic illness in 1992 (3). They discovered that prior hemoglobin injections promptly increased the expression of HO-1 mRNA and protein in the kidney, delaying the progression of renal failure. Since then, Zager and colleagues have shown that HO-1 has a kidney protective effect in a variety of AKI models, including ischemia/reperfusion, glycerol-induced rhabdomyolysis, cisplatin nephrotoxicity, and bilateral ureteral obstruction models (4). They also discovered that blood and urine HO-1 levels rise with the severity of acute renal injury, and the degree of kidney impairment is reduced compared to HO-1 deficiency. Later research has gradually clarified its action mechanism and proved its utility in other chronic kidney illnesses such as diabetic nephropathy, hypertensive nephropathy, glomerular nephritis, and renal transplantation.

## 2 The roles of HO-1

**Figure 1:**
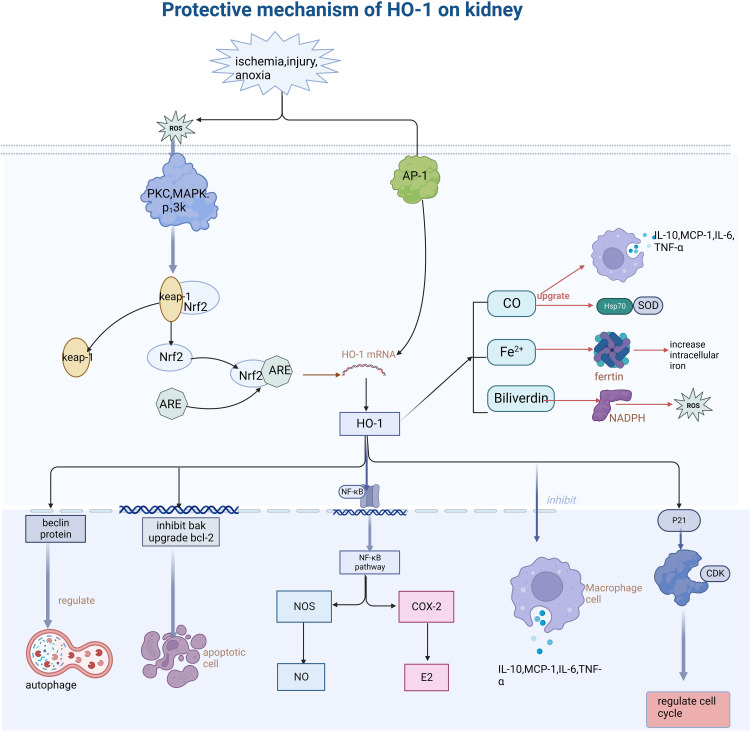
Describes how oxidative stress injury induced HO-1 expression and its function mechanism. Stimulation such as ischemia, anoxic, and injury leads to the generation of ROS, further activates PKC and MAPK protein kinases, dissociates Nrf2 from keap-1, and binds the activated Nrf2 to ARE protein, thus inducing the increase of HO-1 expression. HO-1 promotes the body to recover from injury through various mechanisms such as anti-inflammation, antioxidative stress, cell cycle regulation, anti-apoptosis, etc. (This article introduces its role and mechanism in detail). Secondly, its heme-decomposing products CO, Fe^2+^, and biliverdin also play a role in cell protection by regulating antioxidants, inhibiting iron accumulation in cells, and regulating the secretion of inflammatory mediators. PKC, protein kinase C; MAPK, mitogen- activated protein kinase; AP-1, activator protein 1; Nrf2, NF -E2-related factor 2; ARE, antioxidant response element; Keap-1, Kelch-like ECH- -associated protein-1; NF -kB, nuclear factor-k-gene binding; CDK, Cyclin dependent kinase; ROS, reactive oxygen species; NOS, nitric oxide synthase; COX-2, cyclooxygenase-2.

HO-1 expression in renal cells is induced by ischemia, hypoxia, or stress. It has anti-inflammatory and anti-oxidative functions, modulates cell cycle, inhibits apoptosis, regulates autophagy, influences hemodynamics, and regulates immunity, thus promoting the recovery of kidney tissues and cells from injury (**Figure 1**).

### 2.1 Anti-inflammatory and anti-oxidative stress

The anti-inflammatory action of HO-1 has been supported in various inflammatory models. On the one hand, HO-1 and its by-products can inhibit the production of pro-inflammatory cytokines such as IL-1, IL-6, IL-12, IL-1β, and TNF by activated macrophages, thus playing a role in the macrophage-targeted inflammatory regression stage (5). Up-regulation of HO-1 expression, on the other hand, can limit NF-kB activation and constrain the expression of inflammatory mediators COX-2 and NOS, thus decreasing the generation of COX-2-derived prostaglandin E2 and NOS-derived NO to diminish inflammatory mediator production (6–8). HO-1 knockout mice showed leukocyte macrophage increase, hepatosplenomegaly, and renal tubulointerstitial damage after kidney damage, more inflammatory cell infiltration, and fibrosis in comparison to wild mice accentuated HO-1’s anti-inflammatory action (9).

HO-1 and its metabolites catalyzing the decomposition of heme can play an anti-oxidative role in many ways (10, 11). On the one hand, Heme induces the production of hydrogen peroxide in renal tubular epithelial cells, causing oxidative stress and the production of a large amount of ROS by amplifying lipid peroxidation stress. Secondly, heme inhibits the action of proteasomes such as glutathione reductase, resulting in cell damage and apoptosis. HO-1 inhibits the deleterious effects of heme. This benefit is confirmed by increased heme content and aggravated kidney injury in HO-1 deficient mice (12). Secondly, HO-1 can also reduce the accumulation of intracellular iron, thus reducing the production of oxygen free radicals and peroxide free radicals in cells to inhibit oxidative stress (13). Heme metabolites such as bilirubin and CO have also been shown to inhibit protein phosphorylation and PKC activity, inhibit oxidation of peroxide radicals caused by chemicals, and reduce the production of lipid peroxidation products in tissues and organs, thereby alleviating oxidative stress (14). Bilirubin, in particular, plays an important antioxidant and cytoprotective role in weakening DNA damage and cell death mediated by Ang II (15). CO also promotes the activation of antioxidants such as superoxide dismutase (SOD), NF-kB, and heat shock protein 70 (Hsp70). KD Poss discovered that mice lacking functioning HO-1 were substantially more vulnerable to oxidative stress injury caused by hydrogen peroxide. Anemia resulting from endotoxin injection and the infiltration and fibrosis of renal interstitial inflammatory cells were more visible than in normal mice (10, 16). This also demonstrated that HO-1 upregulation was an adaptive mechanism for protecting cells from stress damage.

Most disease processes are accompanied by inflammation and oxidative stress, so the anti-oxidative stress of HO-1 plays a major role in the protective mechanism of the kidney. Animal studies on renal ischemia-reperfusion injury have shown that the expression of inflammatory mediators such as NF-κB, MCP-1, TGF-β, and other inflammatory mediators is more obvious in HO-1 deficient mice, and the kidney injury is more serious (17), which also indicated that HO-1 could alleviate inflammatory reaction and improve renal tubular injury caused by stress.

### 2.2 Regulation of cell cycle

HO-1 regulates cell cycle progression. The cell cycle is divided into four stages: pre-DNA synthesis (G1), DNA synthesis (S), anaphase (G2), and mitotic phase (M). The progression from one phase to the next is tightly controlled by the production of certain cyclins and the cycle-dependent protein kinase (CDK), which ultimately determines whether the cell cycle is arrested or not. P21 is a member of the Cip/Kip family of CDK inhibitors. Nath and his colleagues were the first to identify the direct regulation of HO-1 on P21 expression on kidney (3): HO-1 regulates P21, limiting target protein phosphorylation and blocking cell cycle progression from G1 to S phase. In addition, CO and iron, the by-products of heme metabolism, also play a role in renal tubular injury and repair through P21-mediated cell cycle arrest. The regulatory effect of HO-1 on P21 has also been found in mesangial cells: HO-1 induced p21 expression in mesangial cells, whereas HO-1 inhibitors counteracted this effect (18).

### 2.3 Inhibiting apoptosis

Heme inhibits the proteasome and causes mitochondrial malfunction, which results in cell damage and apoptosis. HO-1 counteracts this impact by decreasing heme aggregation. HO-1 also inhibits the expression of pro-apoptosis genes like Bak while increasing the expression of anti-apoptosis genes like bcl-2 (19). CO can also prevent apoptosis in fibroblasts and endothelial cells through modulating the MAPK pathway and activating NF-KB in vascular smooth muscle cells, according to recent research (20, 21).

### 2.4 Regulating autophagy

Autophagy is a complex process in which damaged cell structures are transported to the lysosome for destruction and recycling. Autophagy can develop stress adaption and maintain intracellular homeostasis under stress situations such as acute injury and ischemia. Improper autophagy, on the other hand, may contribute to tissue injury. A previous study has shown that HO-1 is an efficient autophagy regulator (22). On the one hand, HO-1 inhibits the production of beclin, a crucial protein involved in autophagy initiation. HO-1 deficient mice showed enhanced autophagy in the proximal tubules, as shown by increased autophagic vesicles and beclin expression in the proximal tubules. On the other hand, a delayed autophagy response may be attributed to the decrease of ROS and heme.

### 2.5 Affect hemodynamics

HO-1 inhibits the level of vasoconstrictor molecules in a tissue-specific manner, regulates the expression of anti-angiogenic CXCL-10, and may alter the key balance between angiogenesis and anti-angiogenesis factors (23). CO also functions as a physiological regulator of vascular tension *via* cGMP-mediated artery response, activating calcium channels and expanding smaller renal arteries. Further study speculated that CO not only relaxes vascular smooth muscles, but also regulates the proliferation and death of smooth muscle cells and may play a role in blood pressure regulation of acute hypertension, which is critical for maintaining the renal microvascular system following damage.

In conclusion, HO-1 alleviates kidney injury after stress injury through the above mechanisms. Recent investigations on kidney transplantation have proven its immune-regulatory effects: HO-1 inhibits the proliferation of T cells and promotes the differentiation of T cells into CD^4+^ CD^25+^ regulatory T cells. It may also participate in the changes in the proportion of immune-related cytokines, and is a key suppressor of B cells and NK cells, which, in conjunction with the foregoing methods, play a role in alleviating kidney damage (24).

## 3 HO-1 in different renal cells

**Figure 2:**
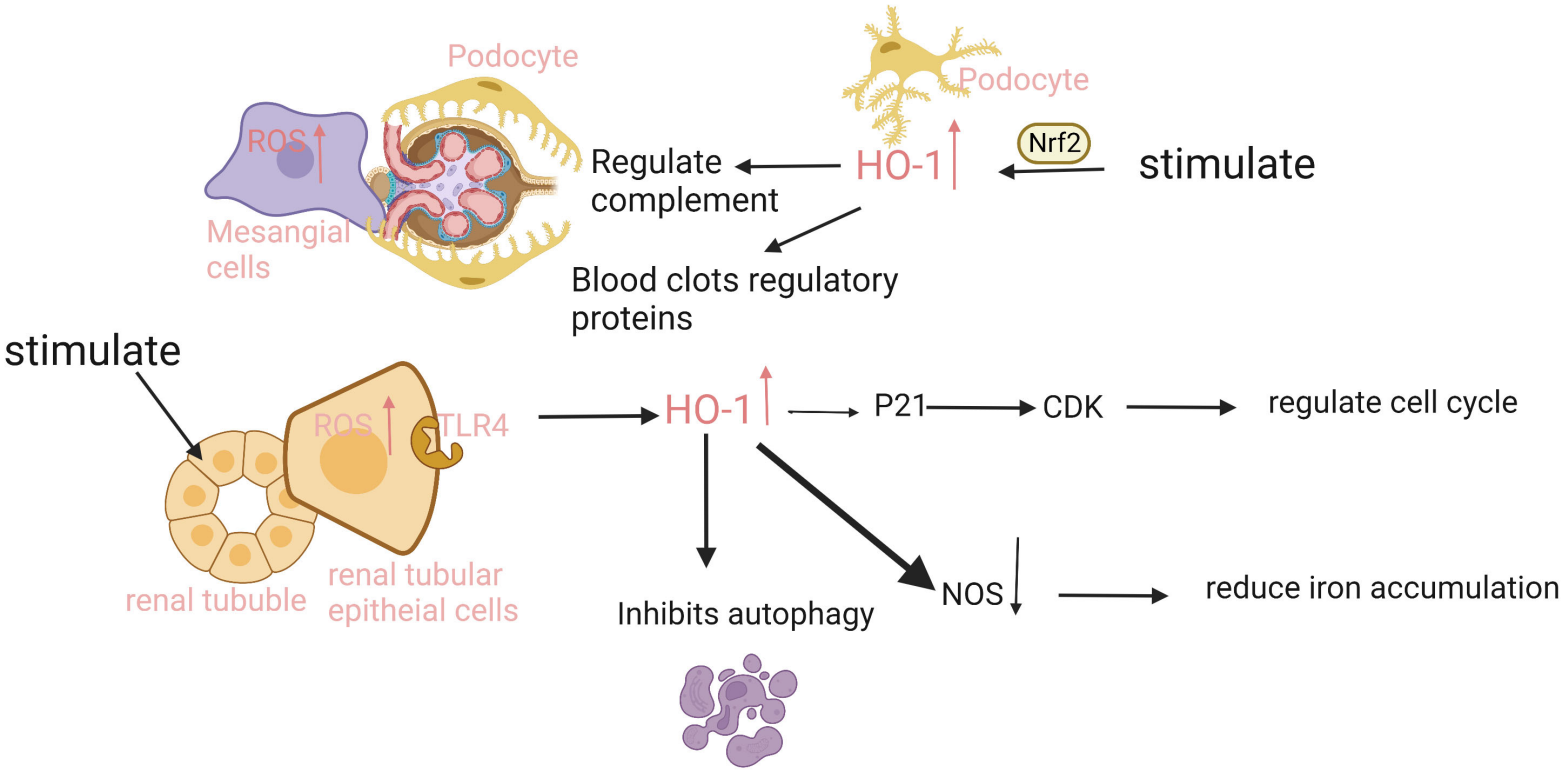
The expression and function of HO-1 is different in different parts of the kidney. The renal tubular epithelial cells have the strongest ability to express HO-1. When injury occurs, ROS in cells increases, and heme binds to the TLR4 receptor on tubule epithelium to promote HO-1 expression. HO-1 upregulates CDK inhibitor P21, reduces tubular cell apoptosis, and inhibits proximal tubular autophagy. In addition, injury can also induce HO-1 expression in podocytes, mesangial cells, and endothelial cells in an Nrf2-dependent manner, regulate complement activation and thrombomodulin, mediate inflammation and thrombosis, and protect cells. ROS, reactive oxygen species; TLR4, Toll-like receptor 4; CDK, Cyclin dependent kinase; NOS, nitric oxide synthase.

HO-1 is expressed differentially in different regions of the kidney, and its functions are outlined in **Table 1**. The mechanism in different kidney cells can be seen in **Figure 2**.

**Table 1:** The roles of HO-1 in renal cells.

Cells	Function	Model	References
Renal tubular epithelial cells	Inhibits autophagy and apoptosis, regulates cell cycle	intravascular hemolysis mice	(25)
The macrophage system	Anti-inflammatory and anti-oxidative stress	Rat allogeneic kidney transplant, diabetic nephropathy mice	(26–28)
Endothelium	Anti-oxidative stress, reduces endothelial injury	Human cases lacking HO-1 HO-1 knockout mice	(29–31)
Podocytes	Adjusts Bax/Bcl - 2 protein ratio, inhibits apoptosis	Diabetic nephropathy rats High glucose podocytes	(32–34)
Glomerular cells	Affects hemodynamics	Glomerulonephritis rats	(35)

Renal tubular epithelial cells, especially proximal tubules, are particularly sensitive to ischemia-reperfusion injury and heme due to the presence of TLR4 (a heme receptor), and the ability to express HO-1 is also the strongest (25). HO-1 regulates constitutive expression of TLR4 in the proximal tubule. It can inhibit autophagy and reduce reactive oxygen species’ accumulation and ferroptosis in renal tubular epithelial cells. Therefore HO-1 can be employed as a key marker to indicate renal tubular damage when the body is severely harmed beyond its adaptive capacity. This is supported by the observation that the renal tubular epithelium is most severely damaged in the HO-1 deficient mouse model of intravascular hemolysis and rhabdomyolysis.

The second is the monocyte-macrophage system, which includes macrophages, dendritic cells, and monocytes (26, 27). Agarwal studied Lewis rats with kidney transplantation and found a significant increase in HO mRNA and protein expression in renal allografts on day 5 after transplantation (28). And the expression of HO protein was most pronounced in the graft’s monocytes, of which more than 80% were macrophages. Their study confirmed the ability of macrophages in the kidney to actively express HO-1 under inflammatory conditions, especially in acute allograft rejection.

Because of their filtering function, renal endothelial cells are the first to be affected by various stressors. Up-regulation of HO-1 in endothelial cells can not only regulate complement activation and thrombomodulin expression, but also inhibit heme-mediated proinflammatory and prothrombotic effects, thereby reducing endothelial injury (29, 30). Endothelial cells in different parts of the kidney, however, differ in their ability to induce HO-1 expression: current evidence suggests that macrovascular endothelial cells are more likely to induce HO-1 upregulation than glomerular endothelial cells, while peritubular capillaries have received little attention (35). Yachie showed that HO-1 is critical for endothelial cell survival by studying the first human example of HO-1 deficiency (31). They found a complete absence of HO-1 production in the tissues of the patient. Glomerular electron microscopy showed glomerular endothelial cell detachment, a large amount of dense material deposited under the endothelium, and severe and persistent endothelial damage in the kidney.

Podocytes are also an important part of the glomerular filtration barrier, which has a filtration function and is particularly sensitive to various damage. The role of HO-1 in podocytes was demonstrated in studies of diabetic rats (32). Vimentin, desmin, and antioxidant proteins are elevated in podocytes during oxidative stress injury, and further upregulates Nrf2 to increase HO-1 production. Heme also stimulated the expression of HO-1 in podocytes in a Nrf2-dependent manner (33). This effect was specifically enhanced in diabetic rat podocytes, and the same results were observed in high-glucose-treated podocytes, implying that this enhancement was associated with the increase of AGEs (34). In glomeruli isolated from diabetic rats, it was observed that the content and activity of HO-1 were significantly increased, the ratio of Bax/Bcl-2 was also increased, and the number of apoptotic cells was obviously increased. Such changes were not observed in Nrf2-deficient rats.

However, the ability of the glomerulus to induce HO-1 is very limited, and infiltrating macrophages rather than glomerular cells are the principal producers of HO-1 in the glomerulus. Mosley investigated heterologous (HNTN) and accelerated (ANTN) nephrotoxic nephritis in Lewis rats, and discovered that HO-1 was not detected by immunohistochemistry in glomerular cells of damaged tissue but was present in cells with macrophage morphology (36).

In short, the expression of HO-1 in the kidneys is highly variable. Renal tubular epithelial cells have the strongest ability to express HO-1 in AKI, and the monocyte-macrophage system cannot be ignored. HO in podocytes is significantly increased in diabetic nephropathy, while its expression in glomeruli is less. However, the reason for its differential expression is still unclear, and it is speculated that it may be related to the site of kidney injury, which needs further exploration.

## 4 Study on the relationship between HO-1 and kidney diseases

The role of HO-1 in kidney disease was first discovered in hemolytic diseases and rhabdomyolysis-associated kidney injury, which was thought to be related to the large release of free hemoglobin. Subsequent studies gradually confirmed its beneficial value in other non-blood-mediated kidney disease (37, 38).

### 4.1 Acute kidney injury

The role of HO-1 in AKI was first discovered by Nath et al. through a glycerol-induced AKI model; they found that a single prior injection of the inducer rapidly induced HO-1 mRNA and protein expression in the kidney, preventing the progression to ARF (3). Follow-up studies were performed in a variety of different models of AKI, such as ischemia-reperfusion injury, hemolytic disease, rhabdomyolysis, cisplatin, and lipopolysaccharide (LPS). The results showed that various stress factors could induce HO-1 RNA and protein expression, which repeatedly confirmed the protective effect of HO-1 on AKI (39).

M J Tracz established a renal IRI model by clamping the renal artery. In this model, it was found that the expression of HO-1 in the kidney, especially in the renal tubules, was significantly increased after renal ischemia-reperfusion injury (23). The renal blood flow of HO-1 knockout mice (-/-) was much lower than that of wild mice at 4 hours after ischemia, and the level of IL-6 was also significantly increased. Subsequent studies further clarified the mechanisms of the protective effects including regulating NO synthesis and hemodynamics, inhibiting apoptosis, anti-inflammation and antioxidant stress factors, etc (40). Shimizu et al. also found a significant increase in HO-1expression in bilateral renal artery ligation-reperfusion rat, and this change was earlier than the increase of serum creatinine concentration (41). However, pretreatment with Sn-MP, a HO-specific competitive inhibitor, aggravated the damage of HO-1 renal tubular epithelial cells in rats.

Rubio-Navarro established a mouse kidney injury model of intravascular hemolytic by injecting the hemolytic drug PHZ. It was found that KIM-1 and NGAL, markers related to renal tubular injury, and HO-1 expression were significantly increased after hemolysis. While Nrf2 deficiency mice did not display an increase in HO-1 expression, KIM-1 and NGAL were higher, and there was extensive proximal tubular necrosis (42). This indicates that HO-1 expression was up-regulated in renal tubules during hemolytic injury and this up-regulation was mediated by Nrf2.

Acute kidney injury caused by trauma-induced rhabdomyolysis, especially crush syndrome, causes a large number of deaths in the ICU. The mechanism of injury includes renal vasoconstriction, oxidative stress, myoglobin participation, and so on. Zager and Johnson found that the contents of TNF-α, MCP-1, and TGF-β1 mRNA increased progressively after glycerol was induced in a mouse model of glycerol-induced rhabdomyolysis. HO-1 increased significantly and reached the maximum after 48 hours of injection (4). The mortality of the mice whose expression of HO-1 was enhanced by a single infusion of hemoglobin was significantly lower than that of the control group. Mice with HO-1 deficiency, meanwhile, were prone to fulminant irreversible AKI, and the mortality rate was almost 100%. The increase of HO-1 expression after rhabdomyolysis-induced AKI can improve renal microcirculation, regulate oxidative stress, protect the kidney, and reduce the risk of death (43).

### 4.2 Sepsis-related renal injury

Sepsis-associated kidney injury is a common renal complication with a high mortality rate in severe patients in the ICU. The beneficial role of HO-1 in sepsis-associated AKI has been gradually clarified in recent years (44, 45). Chung SW et al. (45) established a model of polymicrobial sepsis in HO-1 gene deficient mice and wild-type mice, and found that HO-1 expression increased after sepsis. However, it does not inhibit the generation and accumulation of inflammatory cells, but enhances bacterial clearance by increasing bacterial phagocytosis and the body’s antibacterial response. However, HO-1 gene-deficient mice showed more reduced regional renal blood perfusion and showed higher mortality. Injection of CO-releasing molecules into these mice can increase phagocytosis and alleviate kidney injury in HO-1 deficient mice, which may provide new ideas for further treatment.

### 4.3 Diabetic kidney disease

Diabetic kidney disease (DKD) is one of the major causes of chronic kidney disease and end-stage renal diseases. Previous studies have confirmed the role of HO-1 in high glucose environments (46, 47). A high glucose environment increases the expression of HO-1 in podocytes and up-regulates extracellular SOD and NOS, thus leading to endothelial relaxation and reduction of ROS to affect renal hemodynamics. Inhibition of HO-1 by HO-1 inhibitors such as ZnPP showed an increase in the number of apoptotic cells, which also indicated the protective effect of HO-1. Ahmed et al., using Streptozotocin-induced diabetes as a model in spontaneously hypertensive rats (SHR), found that glomerular albumin permeability was higher and apoptosis was increased in diabetic SHR compared with the control group (48). After induction of HO-1 overexpression by COPP, the permeability of albumin and apoptosis decreased. These results indicated that HO-1 reduces glomerular inflammation and oxidative stress induced by diabetes. HO-1 also has an effect on insulin sensitivity (49); induction of HO-1 expression increased insulin sensitivity, reduced adipose tissue volume, caused adipose tissue remodeling, and improved hyperinsulinism of insulin resistance in diabetes.

The protective role of HO-1 in DKD has also been demonstrated in relevant clinical studies. One study found that HO-1 can be used as an early biomarker of DKD (48). Urinary HO-1 levels were significantly higher in the diabetic nephropathy group than in the non-diabetic nephropathy group, and the increase in urinary HO-1 levels in these patients preceded significant proteinuria. The level of urinary HO-1 is synchronized with the abnormalities of traditional renal function indicators such as creatinine and urea nitrogen. Heme oxygenase and creatinine are jointly involved in the occurrence and development of diabetic nephropathy.

### 4.4 Hypertensive nephropathy

Hypertensive nephropathy is defined as renal insufficiency caused by essential hypertension arteriosclerosis, which is one of the major causes of ESRD. HO-1 is not involved in the maintenance of hypertension under physiological conditions, but plays a key role in the reduction of pathological hypertension, especially Ang-2-dependent hypertension (50). Philip Wenzel found that, in the aorta of Ang-2-induced hypertensive rats, infusion of Ang-2 increased HO-1 expression in aortic adventitia and endothelial cells (51). HO-1-deficient rats were more likely to develop hypertension and cardiac hypertrophy than wild mice. The more extensive and severe the ischemic injury at the cortical-medullary junction, the more obvious the inflammatory mediators and inflammatory cell infiltration in the endothelium. This suggests the expression of HO-1 may decrease the effect of Ang on elevated blood pressure. Sylvia and colleagues established several models of HO-1 specific overexpression, which also confirmed that HO-1 has a specific role in protecting TALH cells from Ang-2 invasion (52). It prevents the increase in Ang2-mediated peroxidase production, significantly decreases prostaglandin E2 levels, decreases blood pressure, and reduces the sensitivity of medullary sodium potassium chloride cotransporter 2 (NKCC2) to furosemide, thereby regulating urinary sodium and attenuating the development of Ang II-dependent hypertensive nephropathy.

### 4.5 Chronic kidney disease

Chronic kidney disease is mainly characterized by renal interstitial fibrosis and glomerulosclerosis. HO-1 inhibits the activation of apoptosis pathway and inhibits macrophage-mediated inflammation and fibrosis progression, thereby inhibiting the loss of periosteal capillaries, inhibiting the activation and proliferation of myofibroblasts, and reducing tubulointerstitial infiltration to delay renal fibrosis (53). The mouse model of ureteral obstruction is a rapid and standard model for studying renal interstitial fibrosis. Correa-Costa demonstrated the ability of HO-1 to prevent and reverse fibrosis formation in CKD through the study of a UUO mouse model (54). They observed the mice treated with ureteral obstruction and found that their glomerular and renal interstitial HO-1 was continuously up-regulated from an early stage. This change occurred earlier than the changes of serum creatinine and urine protein. In HO-1 knockout mice, the fibrosis-related growth factors (such as TGF-β1 and CTGF) and inflammatory factors (TNF-α, IL-1β, IL-6, and IL-10) were significantly higher than those in wild mice. These results suggest that HO-1 can alleviate renal interstitial fibrosis after UUO, inhibit the activation and proliferation of myofibroblasts, and reduce the infiltration of inflammatory cytokines, thereby alleviating renal injury and fibrosis.

### 4.6 Kidney transplantation

Kidney transplantation is currently the best treatment for patients with ESRD, and its technology has been relatively mature. However, transplantation-related ischemia-reperfusion injury, rejection, delayed graft function (DGF), and immunosuppression are still important limiting factors affecting the success rate of transplantation in patients. Reducing renal injury and rejection after transplantation and improving graft function and recipient survival rate are still the research hotspots. As a protective molecule, HO-1 was found to be upregulated in many processes of renal transplantation, including ischemia-reperfusion, DGF, acute and chronic rejection, and xenotransplantation (55, 56). HO-1 improves long-term outcomes after renal transplantation by anti-oxidative stress and inhibiting immune responses. Tullius et al. pretreated donor rats with COPP (a HO-1 inducer) and found that the expression of several inflammatory mediators such as IL-10, IFN-γ, and TNF-α decreased, the polymorphonuclear cell infiltration reduced, and the renal survival rate was significantly improved after transplantation compared with that of unpretreated donor kidneys (57). This effect was observed even if the ischemia time was prolonged to 44h. HO-1 improved the microcirculation of the graft and improved the renal blood perfusion.

HO-1 also plays a vital role in cell proliferation and immune regulation (58): in COPP-treated mice, observed overexpression of HO-1 in the kidney, spleen, and liver were observed. T cell-mediated cellular damage and NK cell-mediated cytotoxicity in the spleen were severely inhibited, and lymphoproliferative hypersensitivity decreased. This indicated that overexpression of HO-1 can lead to inhibition of immune effector cells, which play a vital beneficial role in improving host rejection of grafts during acute rejection. But other studies also showed that HO-1 plays a crucial role in the proliferation and differentiation of B cells: comparing the B lymphocyte of HO-1 knockout mice with those of wild mice, it was found that the B cell counts of HO-1 knockout mice were significantly lower, with growth and development delayed (59). Their results suggested that HO-1 attenuates B cell cycle arrest and apoptosis and stimulates B cell activation and differentiation, which in turn increases the risk of humoral immunity during acute rejection.

In general, many preclinical studies on renal transplantation have shown that induction of HO-1 expression can alleviate graft rejection and attenuate renal injury by inhibiting immune response and oxidative stress. Early HO-1 induction therapy before kidney transplantation is a promising approach to improve renal perfusion and increase graft survival.

HO-1 plays a beneficial role in various acute and chronic kidney diseases and can be used as an early biomarker of AKI and some CKD. Clinical studies have found that the level of HO-1 in the blood and urine of patients with AKI is significantly higher than that of control groups, and it is detectable earlier than the change of serum creatinine and urinary albumin, which can be used as an early marker of AKI (60). A cross-sectional study found that serum HO-1 levels were significantly elevated in sepsis patients with kidney damage, and were positively correlated with sepsis-induced AKI (61). Changes in the level of HO-1 are also seen in CKD such as diabetic nephropathy, which can reflect kidney damage (48).

## 5 Therapeutic potential of HO-1 in renal diseases

A growing body of preclinical evidence has shown that HO-1 plays a beneficial role in multiple kidney diseases through anti-inflammatory, anti-oxidative, anti-fibrosis, and anti-apoptosis mechanisms (62, 63), which has triggered research on the therapeutic value of HO-1 and its inducers in kidney diseases. Whether the HO-1 cell protection pathway can be a promising target for clinical intervention to alleviate kidney injury is an important research field.

A variety of drugs have shown beneficial effects in inducing HO-1 expression and promoting renal protection in preclinical experiments, including heme and its derivatives, metal inducers, pharmaceutical compounds, natural inducers, and HO enzyme by-products. The therapeutic effects are summarized in **Table 2**.

**Table 2:** The therapeutic potential of HO-1 inductors in renal diseases.

Drug		Disease models	Main mechanisms	References
Heme and its analogs	Heme	Rat renal IR	Inhibit pro-inflammatory cytokines, participate in cell metabolism	(36, 64, 65)
	Arginine heme Hemin			
Inducers	CoCl2, SnCl2, ZnCl2,	Mouse IR model	Inhibit inflammatory outbreak, reduce basal NO, improve perfusion	(66, 67)
	COPP	Mouse kidney transplant model,	Inhibit inflammation, inhibit apoptosis, participate in metabolism	(55)
	Natural inducers	mouse model	HO-1 expression	(68)
Drug compounds	Statins	Rat renal IR,	Induce HO-1 *via* the Nrf2 pathway	(30, 69–71)
		aortic smooth muscle cells		
	5-ASA	Mouse colitis model	Induce HO-1 *via* the Nrf2 pathway	(72)
	Celastrol	UUO mouse model	Increase HO-1, anti-inflammatory, inhibit vascular calcification, inhibit fibrosis	(73, 74)
Metabolites of HO	CO	IR mouse model, sepsis mouse model	Anti-inflammatory, regulating P21, inhibiting apoptosis	(75–77)
	Bilirubin	Endotoxemia rats	Inhibit NADPH,ROS	(78)

Heme and its analogues are one of the most potent inducers of HO-1 (36). Pretreatment with a low dose of heme before ischemia can induce HO-1, which leads to a favorable biological response by inhibiting the production of proinflammatory cytokines and producing cytopathic metabolites, thus alleviating renal IRI injury (64). The beneficial effects of arginine heme and hemin on reversing renal oxidative stress injury by upregulating HO-1 in the kidney have also been confirmed in animal experiments, among which arginine heme has been approved for the treatment of acute intermittent purpura in clinic. However, excess heme has prooxidative and lipid peroxidation effects and a risk of thrombophlebitis.

CoCl_2_, SnCl_2_, ZnCl_2_, COPP, and other strong inducers containing metal ions are effective and specific inducers to induce HO-1 expression in the kidney (66, 67). Salom et al. injected CoCl_2_ into IRI rats in advance and found that HO-1 was up-regulated, which further inhibited the outbreak of inflammatory factors, reduced the concentration of basal NO, improved the blood perfusion after ischemia, and relieved renal tubular damage. In the rat ischemia-reperfusion model, similar results were observed when SnCl_2_ was injected intraperitoneally and subcutaneously into mice. In addition, some natural inducers have also shown effects in related studies (68).

Some pharmaceutical compounds such as statins, 5-aminosalicylic acid (5-ASA), paricalcitol, and celastrol can also induce HO-1 expression and reduce tissue damage (30, 69–71):

Tzong-Shyuan Lee et al. conducted *in vitro* experiments on human and rat aortic smooth muscle cells and found that the level of HO-1 was significantly increased in statin-treated cells, with a decrease in inflammatory factors and an increase in the expression of anti-proliferative factors such as p21. The opposite results were detected when treated with HO-1 inhibitors. The effect of 5-ASA on HO-1 induction was found in colitis animal models (72). Palicalcitol mediated HO-1 expression through Nrf2 signal transduction pathway to reduce kidney inflammation and injury (79). Celastrol has previously attracted attention for its anti-inflammatory and anti-cancer effects. Later studies found that it increased the expression of HO-1 in CKD, and dose-dependently inhibited inflammation, vascular calcification, and renal fibrosis (73, 74). This demonstrates its important role in delaying the progression of kidney disease. HO-1 inhibitor could offset its anti-inflammatory and anti-vascular calcification effects.

As an ROS scavenger, the bilirubin and its protective effects have been confirmed. High bilirubin concentration is significantly associated with enhanced endothelial function and reduction of oxidative stress damage (78). As a toxic gas, it was found that a small dose of CO inhalation (1.3 ± 0.2ml/min) before IRI could reveal an increase in HO-1 levels. HO-1 further alleviated renal injury through anti-inflammatory and anti-apoptosis effects, and effectively reduced the chronic fibrosis of kidney and delayed the progression of renal disease (75, 76). Studies in the pig cardiopulmonary bypass model (CPB) also showed that CO inhalation before CPB can improve renal function and reduce tissue damage (77).

However, although preclinical studies on HO-1 and its inducers in the treatment of acute and chronic kidney diseases are abundant and have shown beneficial effects, their side effects are also evident (80): First, in recent years, more and more evidence supports that the excessive upregulation of HO-1 is related to the poor prognosis of certain malignant tumors (81), especially breast cancer, renal cell carcinoma, and liver cancer. It can promote tumor growth, metastasis, and angiogenesis, thus promoting cancer progression. However, it also exhibits the opposite effect in some cancer cells, and its effect on tumors is still controversial. Second, induction of HO-1 improves AKI after IRI. However, most of the relevant preclinical trials are induced by prophylactic administration of HO-1 before the occurrence of ischemia, and the experimental effect is related to the dose and time of administration (82). Its effective therapeutic window is narrow. Some experiments have also found that the application of heme after the occurrence of ischemia can actually promote the progression of injury. Furthermore, the increased expression of HO-1 leads to the accumulation of its by-products such as iron and CO, which further leads to iron-mediated ROS release and CO-mediated vascular effects, aggravating the injury. It may also lead to changes in plasma lipoprotein levels, thereby affecting the susceptibility of the body to atherosclerosis (16, 83). Moreover, some metals and natural inducers have non-specific effects and toxicity, and their safety remains to be confirmed.

The dual role of HO-1 induction therapy and the complexity of the disease hinders the progress from preclinical research to clinical trials. There are still only a few relevant clinical studies. Bharucha et al. confirmed for the first time that the activity and content of HO-1 can be safely induced in humans by infusing hemin into healthy volunteers (65). Small doses of oral D-glyceric acid (DGA) have also been found to upregulate HO-1 and bilirubin in the blood and reduce inflammatory damage in the body. This is especially beneficial to chronic diseases mediated by inflammation (84). However, further clinical research is needed to determine the safety and efficacy of HO-1 induction therapy in humans. Whether long-term HO-1 induction therapy can safely and effectively slow the progression of kidney disease and improve the prognosis needs to be further studied in the future.

## 6 Summary and prospect

Due to the attention and in-depth research on HO-1, its role in kidney diseases has been gradually confirmed. It not only plays a role in heme-mediated pathological diseases, but also participates in various acute and chronic kidney diseases. It is differentially induced in glomeruli, renal tubules, and renal blood vessels to respond to injury and alleviate renal injury through anti-inflammatory and anti-oxidation, immune regulation, apoptosis inhibition, autophagy regulation, and hemodynamic effects. HO-1 induction therapy has shown beneficial effects in preclinical trials, but there are side effects, and relevant clinical research evidence is still insufficient. Several points have been made clear: The expression level of HO-1 affects the progress of kidney disease, and the lack or inhibition of HO-1 will worsen the renal structure and function after injury, while the increased expression has a protective effect; Plasma and urine levels of HO-1 can be used as early biomarkers of certain kidney diseases, earlier than changes in proteinuria and serum creatinine; and HO-1 and its effective inducers can alleviate acute and chronic kidney injury and delay the progression of kidney disease by inducing the expression of HO-1. But HO-1 induction therapy also has many limitations. Inappropriate induction may produce side effects such as toxicity, promotion of cancer, promotion of atherosclerosis, and aggravation of kidney damage. Therefore, the HO-1 enzyme system, as one of the important targets for the treatment of various kidney diseases, provides a new idea for the targeted therapy of kidney diseases. However, it is still in the exploratory stage, and a lot of further research is needed to explore its effectiveness and safety in human applications.

## Author contributions

XW and LN conceived and designed the study. HZ and LN wrote the review article. All authors read and approved the final manuscript.
